# Advances in dynamic visual acuity test research

**DOI:** 10.3389/fneur.2022.1047876

**Published:** 2023-06-23

**Authors:** Ganggang Chen, Jin Zhang, Qi Qiao, Liyuan Zhou, Ying Li, Jie Yang, Jiaxin Wu, Hui Huangfu

**Affiliations:** ^1^Department of Otorhinolaryngology-Head and Neck Surgery, First Hospital of Shanxi Medical University, Taiyuan, China; ^2^Department of Otorhinolaryngology-Head and Neck Surgery, Shaanxi Provincial People's Hospital, Xi'an, China; ^3^Department of Otorhinolaryngology-Head and Neck Surgery, Xijing Hospital of Air Force Military Medical University, Xi'an, China

**Keywords:** dynamic visual acuity test (DVAT), Vestibulo-ocular reflex (VOR), vestibular disorders, dynamic visual acuity (DVA), static visual acuity (SVA)

## Abstract

The dynamic visual acuity test (DVAT) is a functional evaluation tool for the impairment and compensation of the vestibular system, which could reflect the Vestibulo-ocular reflex (VOR) function. We present an overview of DVAT research, displaying recent advances in test methods, application, and influencing factors; and discussing the clinical value of DVAT to provide a reference for clinical application. There are two primary types of DVAT: dynamic-object DVAT and static-object DVAT. For the latter, in addition to the traditional bedside DVAT, there are numerous other approaches, including Computerized DVAT (cDVAT), DVAT on a treadmill, DVAT on a rotary, head thrust DVA (htDVA) and functional head impulse testing (fHIT), gaze shift dynamic visual acuity with walking (gsDVA), translational dynamic visual acuity test (tDVAT), pediatric DVAT. The results of DAVT are affected by subject [occupation, static visual acuity (SVA), age, eyeglass lenses], testing methods, caffeine, and alcohol. DVAT has numerous clinical applications, such as screening for vestibular impairment, assessing vestibular rehabilitation, predicting fall risk, and evaluating ophthalmology-related disorders, vestibular disorders, and central system disorders.

## Introduction

Dynamic visual acuity is the ability to discriminate fine details of dynamic objects during head fixation or in static objects during head or body rotation (1). DVAT is a functional evaluation tool for the impairment and compensation of the vestibular system. DVAT mainly consists of two types: dynamic-object DVAT, in which the observer identifies dynamic objects with a stationary head, and static-object DVAT, in which the observer identifies static objects with a moving head (1). When the head movement frequency is ≥2 Hz, the VOR system is activated (2), which generates compensatory eye movements in the opposite direction of the head movement to maintain stable vision. When the patient has severe vestibular dysfunction or inadequate vestibular compensatory capacity, the visual image slips on the retina, resulting in a loss in dynamic visual acuity, leading to oscillopsia, dizziness, and nausea. This is the fundamental idea underlying the evaluation of vestibular function by DVAT, which can be viewed as a quantitative test of this phenomenon.

## Static visual acuity (SVA)

Visual acuity is the capacity of the eye to distinguish fine details. SVA is defined as the capacity to detect the details of stationary objects whose image is formed on the retina when the subject being evaluated is also stationary. SVA is one of the most frequently used clinical tests for visual acuity. The levels of visual acuity on a visual acuity chart are typically expressed as the Log of the Minimum Angle Resolvable (LogMAR). LogMAR units describe the size of an image based on a ratio of its absolute size to its distance from the eye. Using only SVA to evaluate visual system is inadequate for two primary reasons (3): Many of the visual stimuli to which we must respond in daily life and many sports are frequently in motion; the SVA tests refer to letters or symbols that are commonly exhibited under conditions of maximum contrast (black on white), which is rarely encountered in the various scenarios of everyday life. Therefore, DVA is an essential component of a comprehensive clinical assessment in addition to SVA.

## DVAT methods

Currently, there are various methods of DVAT, but the basic principle remains the same: to access the VOR function by comparing the difference between DVA and SVA. The dynamic visual acuity loss (DVA loss) is calculated as the difference between DVA and SVA.

### Static-object DVAT

#### Bedside DVAT (or non-instrumented DVAT)

Bedside DVAT is a traditional test method, with detailed descriptions reported previously. In brief, the SVA test was performed under static head movement first, using the Snellen visual acuity chart, Landolt C visual acuity chart, or standard logarithmic visual acuity chart. Then the DVA test was performed with the subject rotating his head either actively or by the examiner. The subject's visual acuity was determined to be readable for 50% and above the line of the visual acuity chart, i.e., they could see the lowest line of the visual acuity chart. The DVA loss was calculated by subtracting SVA from DAV. Generally, DVA decreased by no more than three lines on the visual acuity chart compared to SVA or by no more than 0.2 ± 0.08 log MAR; exceeding these ranges often indicates impaired VOR function.

Another method was to ask the subject to identify the optotypes that appear 1 in 1 second when the head is turned. The visual acuity was calculated by the number of correct read optotypes. This method was more sensitive in assessing small changes in visual acuity. However, the traditional DVA test did not specify head movements' amplitude, speed, and frequency. When testing DVA, subjects involuntarily slowed down their head movements to see the optotypes clearly (4, 5), resulting in smooth pursuit rather than eliciting VOR (5). The reliability of the examiner and intra-examiner was also poor for assessing subjects with vestibular hypofunction (6).

#### Computerized DVAT (cDVA)

A computerized DVAT with a rate sensor to control the head velocity and software to control when the optotype is presented, thus increasing its validity (7).

**Figure 1:**
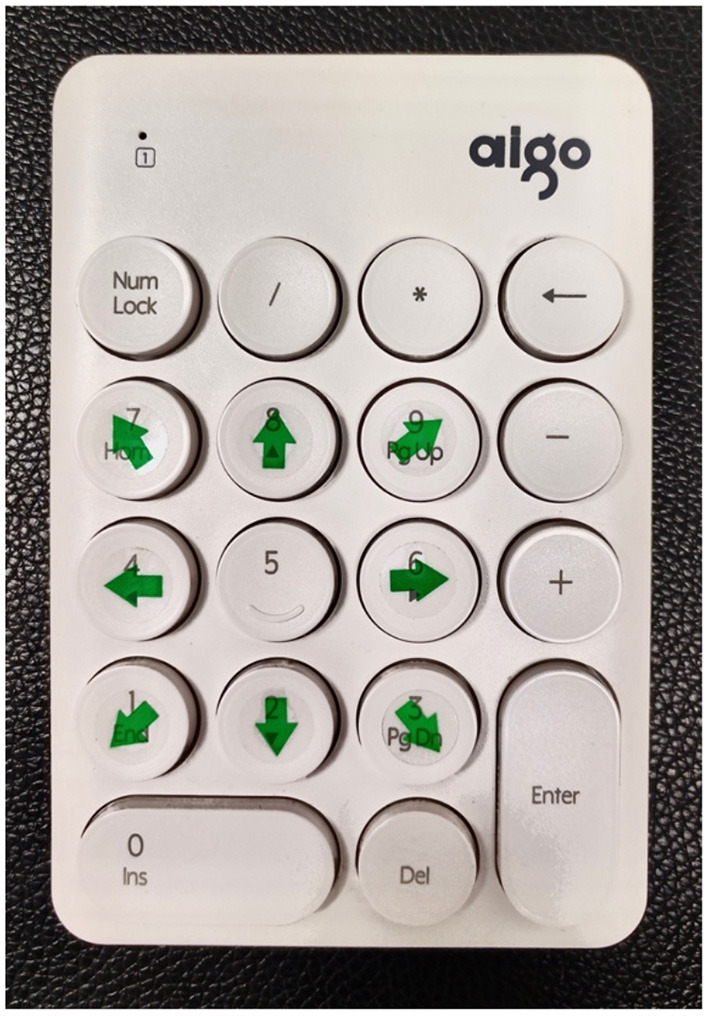
Optotype keypad. Dynamic visual acuity instrument developed by Shanghai ZEHNIT Medical Technology Co., Ltd., Shanghai, China.

**Figure 2:**
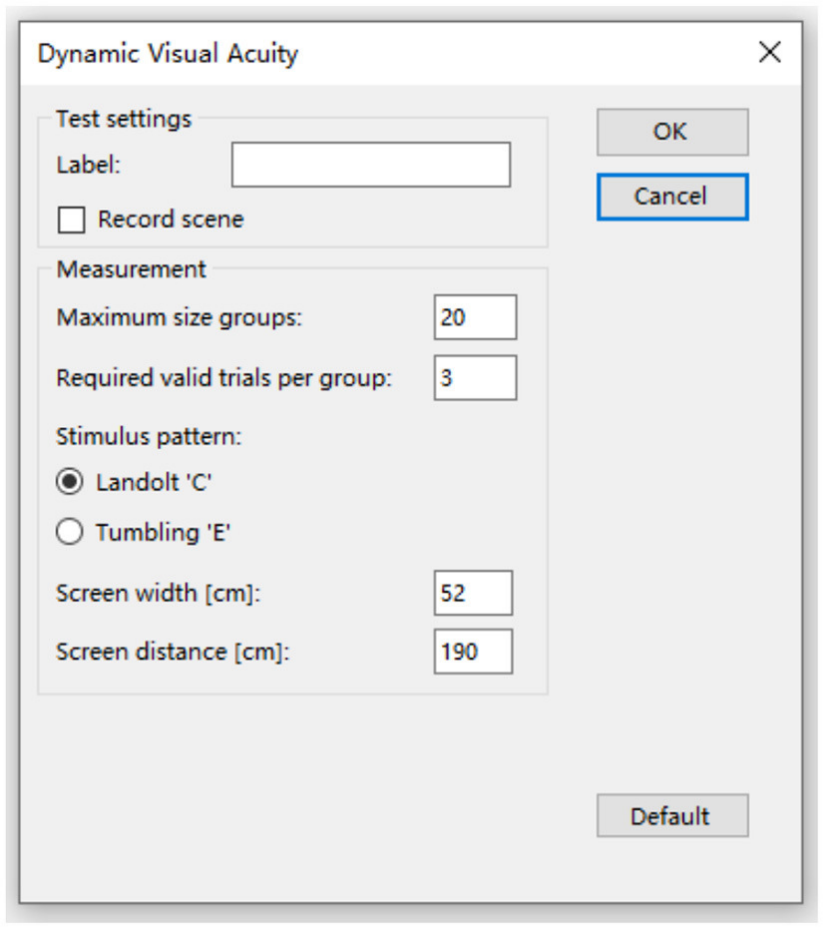
Select the optotype and set the parameters. Dynamic visual acuity instrument developed by Shanghai ZEHNIT Medical Technology Co., Ltd., Shanghai, China.

**Figure 3:**
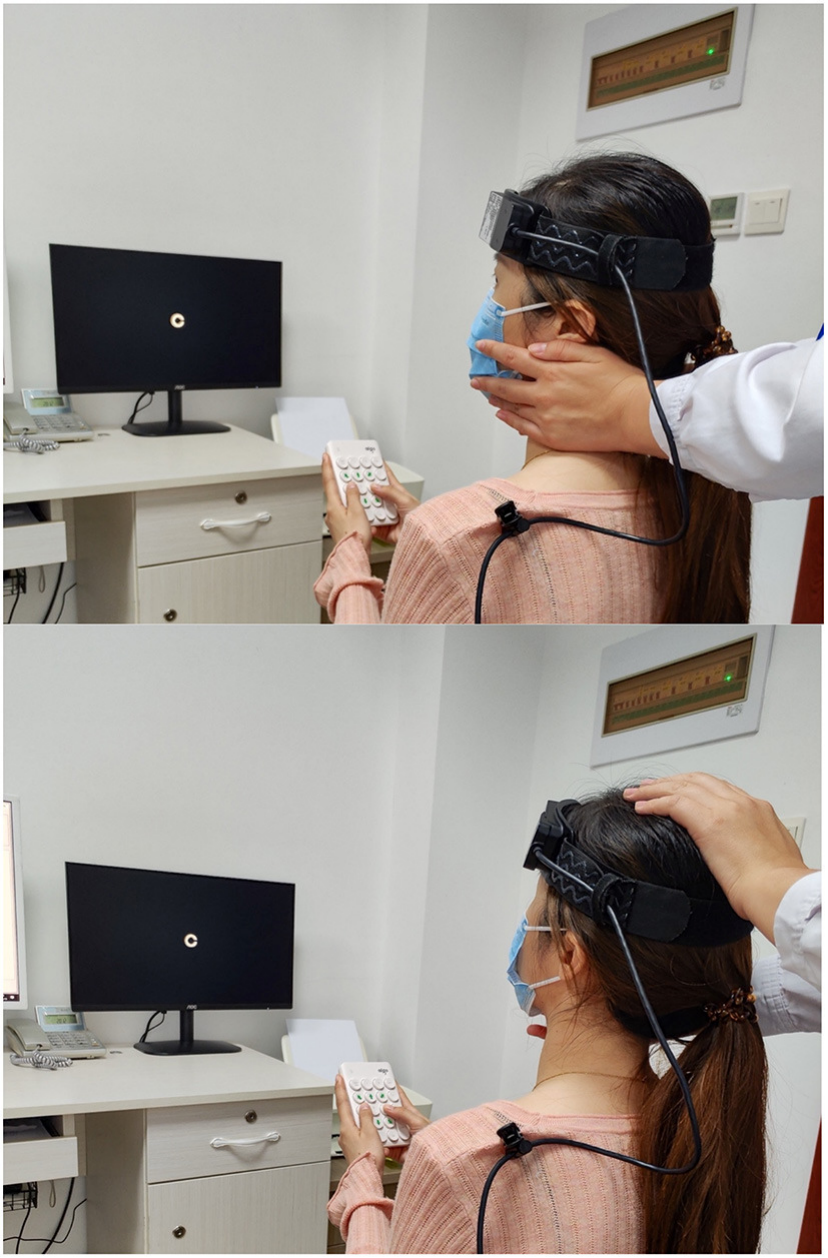
The patient rotated her head to the left/upward passively. Dynamic visual acuity instrument developed by Shanghai ZEHNIT Medical Technology Co., Ltd., Shanghai, China.

When performing cDVA, the subject wore a velocity sensor on his head and held a keypad to select the visual optotype's direction (Figure 1). The optotypes letters “E”/“C” appeared on the computer monitor with different opening directions when the subject performed a sinusoidal movement of his head in the horizontal or vertical direction (Figures 2, 3) with a velocity between 120 and 180°/s for more than 40 ms (8, 9). The optotypes decreased by 0.1 LogMAR per line (5).

The velocity of head movement of the cDVAT test was tolerable for most subjects and avoided the problem of subjects remembering the paper version of the visual acuity chart, which avoided the disadvantages of traditional DVAT and improved the accuracy of the test (10). DVA could be significantly improved by patients' vestibular function rehabilitation (7). So cDVAT can be used to assess the degree of vestibular dysfunction and also to determine rehabilitation treatment goals (5) and has been widely used to evaluate the recovery of gaze stability in patients with unilateral and bilateral vestibular disorders (11).

In a healthy population, the intra-group correlation coefficient (ICC) for cDVAT was 0.87 (>0.75 means better confidence), while in the vestibular dysfunction group, the intra-group correlation coefficient was 0.83 with a sensitivity of 94.5% and specificity of 95.2% (7); the sensitivity of the head during vertical movements was only 37.5% with a specificity of 90% (5).

#### DVAT on a treadmill

Subjects were first tested for SVA while standing on the treadmill and then for DVA while walking at 2, 4, and 6 km/h, respectively. A safety rope attached to the emergency brake of the treadmill was tied around the subject's waist to ensure safety. A chart of Sloan letters (CDHKNORSVZ, ten in total), which consisted of lines of 5 randomly chosen letters, was displayed on a monitor 2.8 m away from the subject. Five randomly selected letters by the computer for each line of sight (the five letters appeared in sequence) if the subject could read two letters accurately and proceeds to the following line (the size of the optotypes is decreased by 0.1 LogMAR).

Subjects were first tested for DVA at a walking speed of 2 km/h, after which they continued to measure DVA at 4 km/h. The test ended when subjects were unable to walk at higher speeds; subjects were excluded if they were unable to walk at 2 km/h on a treadmill (12). It was found that the sensitivity of the DVAT to discriminate patients with bilateral vestibular hypofunction (BVH) was 76% when testing the DVA at a walking speed of 2 km/h only and increased to 97% when measured in combination with speeds of 2, 4, and 6 km/h (13). The DVAT on a treadmill could better reflect the visual acuity change during vertical head movement in daily life. However, the test is challenging for those with BVH and the elderly (12).

#### DVAT on a rotary chair

The DVAT on the rotary chair (10) can be used to detect the effect of the horizontal semicircular on the VOR, which includes a steady-state sinusoidal test and a transient test. The test was performed with the subject's head and torso fixed in the rotary chair at a distance of 6 m. Measurements were made using the letter “E” optotype. A dark red laser dot was used to project the position of the optotype to control gaze when the optotype was not present.

During steady-state testing, the rotary chair was rotated at a frequency of 2Hz sinusoidally at a peak velocity of 10, 20, 40, 70, 100, and 130°/s. The optotype “E” was presented only when the velocity reached 80% of the peak. In transient rotation testing, the subject was rotated at 1,000, 1,600, and 2,800°/s^2^ peak accelerations of the rotary chair. The optotype “E” was presented when the head started to rotate for 50 ms and lasted for 300 ms. The transient rotation test consists of both predictable and unpredictable conditions. For predictable transients, each peak acceleration transmits 40 transients to the right or left in a period of 100 s. For unpredictable transients, there are 60 unpredictable sudden rotations in each direction for each head peak acceleration within a total interval of 150 s, half in each direction. The rotary chair test is not commonly used in clinical practice due to cost and space requirements (14), and the search coil technique and local anesthetic used may cause vision damage during testing (15).

#### Head thrust DVA (htDVA) and functional head impulse testing (fHIT)

Schubert et al. (16) invented a novel method known as htDVA to assess the function of individual semicircular canals. DVA was tested using transient, unpredictable, SCC-plane head thrusts rather than the active head rotation paradigm traditionally used. Some researchers (9, 15, 17–19) used the head impulse testing device-functional test (HITD-FT), also called functional head impulse testing (fHIT), to evaluate the DVA of patients during passive head movements in the horizontal direction (e.g., Figure 3).

Subjects wearing a head-mounted acceleration detector were first tested for SVA. 0.8 LogMAR was then added to the SVA, and subjects were requested to observe the line of the visual acuity chart that was eight lines larger than the SVA, with 10 to 20 effective pulses in both the left and right directions. The subject's head motion amplitude ranged from 10 to 20°, and the pulse direction and timing were randomized to avoid the expected catch-up saccade. When the acceleration reaches 3,000 to 6,000°/s^2^ of effective pulses, the display will show the Landolt C optotypes (the letter “C” has eight directions), which has a duration of 33 ms each time. The fHIT calculates the correct answers (CA%) of the optotypes, which can exceed 98% in normal subjects (9). The test may also be used to plot the curve of head movement and eye movement velocity over time to understand the effect of VOR gain and covert saccade on DVA results. Consequently, its positive detection rate is greater than that of other tests (18, 19).

#### Gaze shift dynamic visual acuity with walking (gsDVA)

The gsDVA test, modified based on the traditional cDVA, now includes a gaze shift component. Chen et al. (11) measured SVA, gsDVA in stance (gsDVAs), and gsDVA with walking (gsDVAw) in healthy subjects and patients with unilateral vestibulopathy (UVH), respectively. The gsDVAw has some significant advantages over the conventional measure of cDVA. The gsDVAs and gsDVAw were measured using three monitors, with the middle monitor appearing randomly as an arrow to the left or right, and the subject turning his head 60° in the direction of the arrow as fast as possible to look at the optotype “E” on the second monitor. The subject then read the direction of the letter as rapidly as possible. The letter's direction remained on the screen until the investigator recorded the response. Once the response was recorded, the letter disappeared from the side screen, signaling the subject to return the gaze to the central display and wait for 2 s before the arrow randomly guided the next head rotation. The gsDVAw was performed by the subject on a treadmill at an appropriate speed, and the rest was similar to the gsDVAs. The gsDVAw tests the patient's visual acuity while walking with head rotation, which is closer to daily life. GsDVAw can distinguish patients with UVH from healthy controls and is regarded as a more realistic measure of gaze stability than the cDVA test.

#### Translational dynamic visual acuity test (tDVAT)

The methods mentioned above are rotational dynamic visual acuity (rDVAT), which assesses the effect of the semicircular canal on the VOR; another test is the translational dynamic visual acuity test (tDVAT), which evaluates the effect of the otolithic organ on the VOR. The tDVAT includes horizontal and vertical forms of movements (20, 21). The patient's body and head are fixed to the examination device, and the test distance is generally within 30 cm, with a horizontal or vertical displacement. Before the optotype on the monitor appears, a “cross” shaped target appears in this location to ensure that the patient is always fixed at the position of the optotype during the movement. A study of healthy subjects showed that tDVA values were worse than rDVA values, and tDVA in the vertical direction was worse than tDVA in the horizontal direction (21).

#### Pediatric DVAT

The pediatric DVAT has been developed and used for years (22–24), showing both reliability and validity. But it has not been studied as wildly as that in adults. Unlike tests for adults, pediatric DVAT typically uses a limited set of letters (H, O, T and V) or common shapes (i.e., Lea vision chart: house, apple, window, ring, circle, square), posted on a 15-line version optotype chart. The positive and negative predictive values, sensitivity, and specificity of pediatric DVAT for detecting children with vestibular hypofunction range from 63 to 100% (22, 25).

The vestibular system may not be developing adequately in children under 6 years of age, which could account for the false-positive results at 2 Hz. But it appears that this interaction is mature enough to be exploited in a walking task by the age of five. Vervecque et al. (26) found DVAT on a treadmill useful for preschoolers of age 5. Rine et al. (23) showed that hDVA is reliable for children as young as 3 years, with excellent screening for vestibular hypofunction. In addition, it is interesting to note that DVA in children with sensorineural hearing, and children with cochlear implants were also decreased (27, 28).

### Dynamic-object DVAT

Redondo et al. (29) examined subjects with heads stationary to look at a monitor 4 m away and measured the DVA for two conditions: the horizontal left-to-right sliding of the optotype “E” at 5, 10, 20, 30°/s, and random appearance (random Brownian motion). Each optotype was displayed for a maximum of 20 s during the test. Optotypes of the same size were measured five times, beginning with letters of 0.8 LogMAR in size. When the subject had three correct identifications, the optotypes decreased by 0.1 LogMAR; when <3, the visual acuity corresponding to that optotype was the subject's final visual acuity.

Another study (30) designed a novel DVA system capable of measuring DVA in the presence of predictable, random, and jittering target motion. When measuring DVA, the optotypes on the monitor were presented for a maximum of 16 s with horizontal, vertical, oblique, or random movement. This DVA system was shown to closely agree with the early treatment diabetic retinopathy study (ETDRS)visual acuity chart (ICC = 0.726), and the retest reliability was good.

The Dynamic-object DVAT mainly reflects a smooth pursuit ability and is commonly used for visual acuity evaluation of some athletes tracking balls.

## Clinical applications

### Vestibular disorders assessment

DVAT can be used to assess vestibular disease by stimulating the peripheral vestibular organs and the corresponding signaling pathways, where impairment of these can lead to a decrease in DVA. The DVAT evaluation of UVH, Benign Paroxysmal Positional Vertigo (BPPV), and vestibular neuritis (VN) is based on this principle.

#### Vestibular hypofunction

In some related studies (5, 11), for UVH patients (*n* = 168), the affected side DVA was worse than the healthy side (*p* < 0.001), while the asymmetry was not found in most BVH patients; secondly, both UVH and BVH patients had worse DVA than normal subjects. As a result, this asymmetry may help to distinguish between UVH and BVH patients, as well as the healthy/affected side of UVH patients.

#### BPPV

A study (31) evaluating DVA in horizontal and vertical directions in patients with horizontal and posterior semicircular BPPV found that BPPV patients had worse DVA than healthy subjects (*p* < 0.01). However, it is unclear whether this was due to vertigo symptoms or abnormal vestibular function. Neither horizontal nor vertical DVAT could distinguish between the affected and healthy sides of the patients.

#### VN

The study by Viciana et al. (32), which included patients with unilateral vestibular neuritis and healthy subjects, evaluated the three semicircular canal functions using htDVA in the horizontal and vertical directions, respectively. The results indicate that htDVA has low sensitivity (22%) and high specificity (85%), making it potentially helpful in monitoring vestibular rehabilitation and DVA in patients with VN.

### Ophthalmic diseases assessment

Currently, DVATs are used to evaluate patients with ophthalmic diseases such as cataracts, optic neuritis, and glaucoma (33).

#### Cataract

Cataract is a prevalent ocular disorder with a cloudy area in the lens of the eye that leads to vision loss. Several studies (34, 35) have shown that dynamic visual motion significantly impacts age-related cataract patients, and DVA can be remarkably improved after phacoemulsification combined with Intraocular lens (IOL) implantation surgery. It has more significant advantages over traditional SVAT in evaluating the visual function during driving and exercising.

#### Optic neuritis

Optic neuritis is one of the most prevalent clinical features in multiple sclerosis that results in acute visual acuity decrease. The demyelination of optic nerves causing reduced projection rate along the visual pathways might be detected by DVAT (36). As compared with SVAT, DVAT may be more appropriate to quantify projection latencies caused by demyelination because the generation of DVA requires a sufficient amount and velocity of visual input projection, while SVA only depends on the amount (36, 37).

#### Glaucoma

Glaucoma is characterized by progressive degeneration and death of retinal ganglion cells. The moving-optotypes DVAT using high temporal frequency optotypes has potential clinical value in the earlier detection of functional defects in glaucoma (38, 39).

Finally, it is essential to note that the current study showed that htDVA does not help diagnose superior canal dehiscence syndrome (SCDS) (8). Janky et al. (8) showed that SVA and active DVA are not significantly affected in SCDS patients after surgical canal plugging. However, postoperative htDVA was reduced considerably in the SC plane on the affected side, and this reduction persisted beyond 6 weeks. This may be a permanent effect of surgical occlusion.

### Evaluation of central system disorders

DVAT can assess central system disorders such as cerebral concussion, multiple sclerosis (MS), and cerebellar ataxia (CA).

#### Cerebral concussion

Gottshall et al. (40) determined that patients with acute traumatic brain injury (TBI) scored higher on DVAT compared to healthy controls. They recommended the DVA test as an outcome measurement tool in assessing TBI patients.

Athletes are often prone to concussions from intense exercise and should undergo DVAT early to help initiate vestibular rehabilitation exercises for patients as soon as possible to avoid delayed recovery (41). Pediatric patients with concussions require longer recovery times compared to adults. Zhou and Brodsky (42) found DVA abnormalities in 57% of children with sports-related concussions with dizziness and balance disturbances, which suggests that high-frequency VOR injury may be the primary cause of dizziness in children.

#### MS

About 70% of MS patients may have brainstem injury, and 87% have abnormal brainstem reflexes (43), leading to abnormalities in their VOR mediated by brainstem pathways and thus abnormal DVA. Compared to healthy controls of the same age, dDVAT levels in MS patients were 2.5 times worse (44). Mañago et al. (43) evaluated MS patients using GST with cDVAT. It was found that both tests could distinguish MS patients from the healthy population, but the cDVAT was unable to distinguish the degree of functional impairment in MS patients.

#### CA

One study (45) found a decrease in DVA of up to 84% in CA patients, mainly associated with impaired VOR, and only marginally to the degree of ataxia. CA patients with concomitant vestibular impairment showed a similar decrease in DVA as BVH patients. However, DVA impairment was also seen in patients with CA who lacked a severe vestibular lesion (45), indicating the involvement of central mechanisms such as the impairment of central adaption of VOR. The evaluation of DVA in patients with progressive CA could offer information to target vestibular and oculomotor rehabilitation.

#### Migraine

A recent study (46) found that migraine patients had significant DVA loss as compared with control subjects in four positions (left DVA, right DVA, up DVA, and down DVA, respectively. These abnormal DVA findings may be due to impaired VOR reflex and visuo-vestibular cortical interactions, which could explain the pathophysiology of head movement hypersensitivity and visual motion sensitivity encountered by migraine patients. Given this finding, oculomotor and gaze stability exercises that improve VOR gain may be a promising therapeutic option that could decrease head movement hypersensitivity and visual motion sensitivity and reduce the frequency of migraine attacks.

### Evaluation of the effects of vestibular rehabilitation

The DVAT is frequently used to evaluate the effectiveness of vestibular rehabilitation in patients during active head movements (17, 47). UVH patients who underwent vestibular rehabilitation exercises showed a significant improvement in DVA during active head movements (*P* < 0.01), whereas patients who underwent placebo exercises showed no improvement (*P* = 0.07) (48). Vestibular rehabilitation exercises also improved DVA in patients with BVH (5). Additionally, fHIT was also used to assess compensations of the acute phase and 3 months after the onset of VN (19). Several studies have also used cDVAT to evaluate the effect of gaze stabilization exercises (49).

In addition, DVAT can also assess the effect of the vestibular implant (VI) on gaze stabilization in BVH patients. According to one study (50), when the vestibular implant system was open, the difference between SVA and DVA in BVH patients was 0-0.16 LogMAR. This implies that DVA can approach normal values in patients with VI and indicates that VI has good prospects for application (50). Because of the close relationship between DVA and ball sports (e.g., basketball, soccer, volleyball, table tennis, etc.), DVA is also commonly used to evaluate the effect of physical activity on improving visual acuity (1).

### Prediction of fall risk

The coordination of visual, proprioceptive, and vestibular senses is essential for maintaining body balance. When the VOR pathway is impaired, patients especially those with BVH, may experience oscillopsia and be at risk of falling if they rely only on proprioception (51). Age-related decline in balance function is accompanied by vestibular impairment, seen in three-quarters of seniors who needed fall risk assessments. This suggests that vestibular impairment may contribute to the risk of falling in the elderly (52).

Hall et al. (51) combined the dynamic gait index (DGI) and DVA scores to construct a model that could predict the risk of falls in patients with UVH. The patient's DGI and DVA scores were brought into the formula to derive the patient's fall risk, and the sensitivity and specificity of the model were 77 and 90%, respectively. Honaker and Shepard (53) used the DGI score (DGI ≤ 19 as susceptible to falls) as the gold standard in a screening study of 16 patients with a history of falls. When the DVAT value was >0.25 LogMAR, it suggested the need to assess patients for fall risk with a sensitivity and specificity of 92 and 61%, respectively. Bayan et al. (47, 54) also suggested the DVAT as an additional means of assessing the risk of falls in patients with mild cognitive impairment.

## Influencing factors

### Subjects

#### Age

The relationship between age and DVA is unclear. It has been suggested that age has a negative effect on DVA in both healthy people and patients with vestibular dysfunction, i.e., the DVA will get worse as one gets older (5, 7, 8, 32, 55–57) and that DVA is more degenerated than SVA (58). It has also been suggested that DVA remains stable until the age of 50 and begins to decline after that (24, 59). The possible reason for this is the physiological degeneration of neuronal function in the vestibular nuclear complex (59). However, other studies did not find a correlation between age and DVA in patients with UVH/BVH (11, 55). Therefore, the relationship between age and DVA in patients with vestibular dysfunction needs further investigation.

#### Occupations

In one study, the DVA of water polo players (58) and soccer players (60) was compared to the DVA of the general population and showed that the DVA of athletes was better than that of the general population. Additionally, the DVA varies between occupations and even within the same occupation. For instance, water polo players have a DVA of about 0.5 (61), while among soccer players, goalkeepers have the best DVA (0.82) and strikers have the worst DVA (0.62).

#### SVA

Some studies suggested that DVA is possibly dependent on SVA. This might be related to the difference in focusing with different SVA. Poorer SVA can cause the defocus of the retinal image, which affects DVA (62). SVA is mainly related to the power of ocular resolution, while DVA is also closely related to the functionality of the oculomotor system. Nakatsuka et al. (62) examined 42 subjects with normal visual acuity and demonstrated a strong correlation between SVA and DVA (*r* = 0.87, *P* < 0.001).

However, the correlation between DVA and SVA was not significant, according to a different study (63), probably because SVA is related to the discriminative ability of the eye, whereas DVA is related to VOR function. It is common to find significant individual differences in DVA in subjects with similar SVA (64).

Weissman and Freeburne (65) examined the relationship between DVA and SVA in 30 female college students with Landolt C scopes at six speeds (20, 60, 90, 120, 150, and 180°/s). The results showed a significant linear relationship between DVA and SVA at the first four speeds (*P* < 0.01). However, the distribution range of SVA was different, and the linear relationship between the two was different; the linear relationship between the two disappeared at the latter two speeds (*P* > 0.09). The correlation between DVA and SVA is usually low and inversely proportional to the speed of stimulation. Therefore, the different conclusions reached by the various studies may be due to the different speeds of the movement.

#### Subjects' eyeglass lenses

Eyeglass correction may have an impact on DVA because of peripheral defocus and prism effects, which result in unclear and skewed images in the peripheral region. The subjects' DVA declined with increasing diopter (66, 67). Multifocal contact lenses reduced these effects, resulting in better DVA in subjects compared to regular glasses, but the difference was not statistically significant (*p* = 0.4) (67).

### Testing methods

Several studies (5, 17, 48, 68) have indicated that patients with active htDVAT have higher DVA values than those with passive htDVAT. This might be due to the activation of the cervico-ocular reflex when patients actively perform head movements (15) and a shorter latency for the appearance of covert saccades, which reduces the slipping time of the optotype on the retina (17, 68, 69). As a result, some researchers have suggested that measurements performing passive head rotation are closer to the true DVA values (13, 16, 21), while others have suggested that active head rotation in patients is more consistent with daily life (17). Therefore, some researchers believe that measurements during passive head rotation of patients are closer to the true DVA values (13, 16, 21). In contrast, others believe that measures during active head rotation of patients are more comparable to daily life (17). In addition, because of saccade suppression, the shorter the appearance time of the optotype on the monitor, and the faster it moves, the worse the DVA results. The linear relationship between visual acuity and angular velocity of the optotype is Y = a + bX (30, 58). The time of optotype appearance has a more significant effect on DVA compared to the movement velocity (63). Regarding movement track, horizontal movement of the optotype gets better DVA results compared to tilted movement (58).

Roberts and Gans (70) showed that the sensitivity and specificity of the vertical DVAT (vDVAT) were 42.4 and 93.8%, respectively, and the horizontal DVAT (hDVAT) were 66.7 and 86.2%, respectively. However, the accuracy of these two DVAs did not have a significant difference, 76.5 and 79.6%, respectively (70). Another study revealed that vDVAT was <55% accurate in identifying patients with abnormal vestibular function vs. those with dizziness but normal vestibular function (with 23.1% accuracy for UVH and 54.5% accuracy for BVH), whereas hDVAT was more than 90% accurate in identifying both (with 93.1% accuracy for UVH and 96.1% accuracy for BVH) (59). The difference between vDVA and hDVA may relate to the detection of the acceleration signal. Both studies suggest that hDVAT is more sensitive and accurate compared to vDVAT. The reason for this may be that the bilateral vertical semicircular canals are able to complement each other during vDVA testing, making it easier to maintain visual field stability and thus less likely to detect dysfunction. In contrast, only the unilateral horizontal semicircular canals perceive head motion acceleration during hDVA testing, which is not conducive to maintaining visual field stability, thus making it simpler to detect dysfunction on the affected side (59).

### Caffeine and alcohol

Caffeine also influences vision, as it increases eye movement speed and contrast sensitivity (71). A randomized controlled study analyzed the effects of caffeine on DVA (29). The study was conducted on a population with low levels of coffee intake (2 cups of espresso per day). The test and control groups consumed 4 mg/kg of caffeine and placebo, respectively, and were then measured for DVA after 60 min of consumption, i.e., the acute effects of caffeine on DVA. The study measured the visual acuity and reaction time of the subjects and found that caffeine consumption increased the DVA values in horizontal and random directions and shortened the reaction time of the subjects to horizontal motion optotypes in a short period of time. However, the effect of high levels of caffeine intake on DVA has not been studied.

Alcohol affects the nervous system and cognitive function. The degree of DVA loss in subjects after alcohol intake increases, although SVA remains unchanged (72). This is associated with reduced responsiveness of the oculomotor and vestibular systems after alcohol intake, especially the reduction of VOR function.

## Discussion

DVAT is a safe and effective screening method for VOR function. Studies have demonstrated that the DVAT can be used to screen for vestibular impairment, assess vestibular rehabilitation, assess ophthalmology-related disorders, evaluate central system disorders, and screen athletes and pilots. DVAT can also be complemented with other vestibular function tests for a comprehensive evaluation. As a diagnostic tool, the DVA test should be used with caution since the test score reflects central compensation for vestibular dysfunction and, therefore, is not a pure measure of peripheral vestibular function.

Nowadays, clinicians increasingly realize the significance of the DVAT, which was recommended as a standard diagnostic evaluation tool for vestibular diseases in some worldwide clinical guidelines and consensus (73, 74), and was chosen by the NIH Toolbox as an assessment of the vestibular system's contribution to gaze stability (23).

However, there is still a lack of studies with large samples to define DVAT's criteria, application indications, abnormal values, and specific applications in different diseases. The prospective clinical applications of DVAT are expansive.

Future studies on the role of DVAT in the diagnosis and treatment of various diseases are needed to explore more effective testing methods and better clinical applications.

## Author contributions

GC: study concept and design, study supervision, and manuscript writing. JZ: study concept and design, collection and analysis of relevant literature, and manuscript writing and revision. QQ: collection and analysis of relevant literature and manuscript writing. LZ: manuscript reviewing and manuscript revision. YL: study concept and design and collection and analysis of relevant literature. JY and JW: collection and analysis of relevant literature and create figures. HH: critical revision of the manuscript for important intellectual content. All authors contributed to the article and approved the submitted version.

## Funding

This work was supported by Shanxi Province Medical Key Scientific Research Project (2020XM13) from Shanxi Provincial Health Commission.

## Conflict of interest

The authors declare that the research was conducted in the absence of any commercial or financial relationships that could be construed as a potential conflict of interest.

## Publisher's note

All claims expressed in this article are solely those of the authors and do not necessarily represent those of their affiliated organizations, or those of the publisher, the editors and the reviewers. Any product that may be evaluated in this article, or claim that may be made by its manufacturer, is not guaranteed or endorsed by the publisher.
